# PEDF Is Associated with the Termination of Chondrocyte Phenotype and Catabolism of Cartilage Tissue

**DOI:** 10.1155/2017/7183516

**Published:** 2017-01-16

**Authors:** P. Klinger, S. Lukassen, F. Ferrazzi, A. B. Ekici, T. Hotfiel, B. Swoboda, T. Aigner, K. Gelse

**Affiliations:** ^1^Department of Orthopaedic and Trauma Surgery, University Hospital Erlangen, Erlangen, Germany; ^2^Institute of Human Genetics, Friedrich-Alexander-Universität Erlangen-Nürnberg, Erlangen, Germany; ^3^Division of Orthopaedic Rheumatology, Department of Orthopaedics, University of Erlangen-Nürnberg, Erlangen, Germany; ^4^Department of Pathology, Medical Center Coburg, Coburg, Germany

## Abstract

*Objective.* To investigate the expression and target genes of pigment epithelium-derived factor (PEDF) in cartilage and chondrocytes, respectively.* Methods.* We analyzed the expression pattern of PEDF in different human cartilaginous tissues including articular cartilage, osteophytic cartilage, and fetal epiphyseal and growth plate cartilage, by immunohistochemistry and quantitative real-time (qRT) PCR. Transcriptome analysis after stimulation of human articular chondrocytes with rhPEDF was performed by RNA sequencing (RNA-Seq) and confirmed by qRT-PCR.* Results.* Immunohistochemically, PEDF could be detected in transient cartilaginous tissue that is prone to undergo endochondral ossification, including epiphyseal cartilage, growth plate cartilage, and osteophytic cartilage. In contrast, PEDF was hardly detected in healthy articular cartilage and in the superficial zone of epiphyses, regions that are characterized by a permanent stable chondrocyte phenotype. RNA-Seq analysis and qRT-PCR demonstrated that rhPEDF significantly induced the expression of a number of matrix-degrading factors including SAA1, MMP1, MMP3, and MMP13. Simultaneously, a number of cartilage-specific genes including COL2A1, COL9A2, COMP, and LECT were among the most significantly downregulated genes.* Conclusions.* PEDF represents a marker for transient cartilage during all neonatal and postnatal developmental stages and promotes the termination of cartilage tissue by upregulation of matrix-degrading factors and downregulation of cartilage-specific genes. These data provide the basis for novel strategies to stabilize the phenotype of articular cartilage and prevent its degradation.

## 1. Introduction

Pigment epithelium-derived factor (PEDF), also known as Serpin F1 (SERPINF1), was originally isolated from human fetal retinal pigment epithelial cells and was defined as a potent antiangiogenic factor [1, 2]. PEDF is also expressed in a multitude of other tissues and represents a multifunctional protein that also includes neurotrophic, neuroprotective, and antitumorigenic properties [3–7]. PEDF is also present in the skeletal system, in particular in regions undergoing endochondral ossification and bone remodeling [5, 8, 9]. During mouse development, PEDF is expressed in chondrocytes within epiphyseal growth plate cartilage [10]. PEDF could also be found in the growth zone of the deer antler [11]. While healthy articular cartilage typically lacks PEDF, it is detectable in osteoarthritic cartilage [9, 12]. High levels of PEDF could also be detected in osteophytes [9], which suggests a role of PEDF in terminal chondrocyte differentiation and the endochondral ossification process. Recently, mutations of the SERPINF1-gene resulting in loss-of-function of PEDF cause the recessive osteogenesis imperfecta type IV that is characterized by a severe skeletal phenotype with increased risk of fractures and skeletal deformities [13–15]. But the role of PEDF within skeletal tissues is still not clear. PEDF was suggested to support mineral deposition by induction of osteoblastic genes [16]. In osteosarcoma cells and mesenchymal progenitor cells, PEDF was shown to exert antiangiogenic and proapoptotic effects involving upregulation of FasL [9, 17]. However, differentiated chondrocytes were not permissive to PEDF-induced apoptosis [9], which suggests further functions of PEDF within the skeletal tissue.

Thus, the aim of this study was to investigate the functional role and involved genes of PEDF in cartilaginous tissue, in particular during the process of endochondral ossification. Therefore, we focused on the distribution of PEDF within different human cartilaginous tissues of different developmental stages that are subjected to endochondral ossification, including the fetal epiphysis, the postnatal growth plate, and adult articular and osteophytic cartilage. As a reference, MMP13 expression served as marker for endochondral ossification. Transcriptome analyses using RNA sequencing (RNA-Seq) and bioinformatics pathway analyses based on PEDF-stimulated human articular chondrocytes were performed to define potential target genes and downstream-pathways activated by PEDF.

## 2. Methods

### 2.1. Tissue Samples

Human epiphyses (20-week gestation) and postnatal epiphyseal growth plate cartilage were obtained from donors at autopsy. Human adult articular cartilage as well as osteochondral osteophyte samples was obtained as matched pairs from the same knee joints of 10 patients undergoing total knee arthroplasty for osteoarthritis (mean age 75.5 years, range 70–82 years). The diagnosis of primary osteoarthritis was based on clinical and radiographic evaluations according to standard criteria. Secondary osteoarthritis or rheumatoid arthritis was excluded from this study. For mRNA expression analysis, macroscopically intact articular cartilage (AC) was isolated from osteochondral specimen originating from the resected femoral condyles with a macroscopically intact joint surface characterized by an Outerbridge score of 0 or 1. Osteophytic cartilage (OC), as well as periosteal mesenchyme (PM) and subchondral bone (B), were isolated from osteochondral samples of the same respective joints originating from the edges of the femoral condyles. Only joints with distinct osteophyte formation were used for this study. Osteophytes were distinguished from the marginal transition area of the joint surface by the existence of a concave ridge towards the joint surface.

For protein isolation and mRNA-analysis, articular cartilage, osteophytic cartilage, periosteal mesenchyme, and bone were dissected and isolated. In order to distinguish between articular cartilage and to selectively isolate the cartilaginous cap of the osteophytes and to exclude any abrasion of bone trabeculae and calcified tissue of the deepest cartilaginous zone, only minimal forces were applied with a scalpel by cutting tangentially to the surface to yield thin cartilaginous osteophyte slices of less than 1 mm thickness. Each patient gave informed consent prior to surgery, and the institutional ethics committee approved the study (Ref. number 3555).

### 2.2. Immunohistological Analysis

Specimens of human fetal epiphyses, human fetal epiphyseal growth plates, and human adult osteochondral section from the edges of the femoral condyles, containing periosteal mesenchyme, osteophyte cartilage, articular cartilage and subchondral bone, were used for histological analysis. All osteochondral specimens were fixed in 4% paraformaldehyde for 12 hours, followed by decalcification in 0.5 M ethylene diamine tetraacetic acid (EDTA) for 3 months. After standard processing, the samples were embedded in paraffin. Specimens were cut in serial transverse 5 *µ*m sections and stained with toluidine blue (TB) or hematoxylin/eosin (H/E) for morphological assessment.

For immunohistochemical detection of PEDF, sections were pretreated with 0.05% trypsin and incubated with goat anti-human PEDF antibodies (Santa Cruz Biotechnology, Santa Cruz, CA). For detection of MMP13, sections were pretreated with 0.05 U/ml Chondroitinase (Sigma-Aldrich, Taufkirchen, Germany) and incubated with mouse anti-human MMP13 antibodies (Calbiochem, Darmstadt, Germany). Negative control sections were incubated with isotype normal mouse IgG (Santa Cruz Biotechnology). The sections were incubated with biotinylated anti-goat or anti-mouse secondary antibodies (Dianova, Hamburg, Germany), respectively. Bound antibodies were visualized by a complex of streptavidin and biotinylated alkaline phosphatase (Vectastain, ABC-AP, Vector Laboratories, Burlingame, CA). The sections were developed with fast red and counterstained with hematoxylin. Immunohistochemical detection of type I and type II collagen was performed as previously described in detail [18].

### 2.3. Immunoblot Analysis

Protein extracts were obtained from articular cartilage, osteophyte cartilage, and bone as described previously [19]. After boiling, 50 *μ*g protein extracts were separated by sodium dodecyl sulfate (SDS) PAGE on a 10% gel and transferred on nitrocellulose transfer membranes. The efficiency of protein transfer was controlled by Ponceau S staining. After blocking, the nitrocellulose membranes were incubated with a monoclonal mouse anti-PEDF antibody diluted 1 : 1250 (Abcam, Cambridge, UK) or a rat anti-*α*-tubulin antibody (diluted 1 : 1000) (AbD Serotec, Kidlington, UK) overnight at 4°C. Horseradish peroxidase-conjugated goat anti-mouse antibodies (GE Healthcare, Little Chalfont, UK) or goat anti-rat antibodies (Dianova, Hamburg, Germany) (diluted 1 : 10000) were used as secondary antibodies. The immunoreactive proteins were visualized using a chemiluminescence kit (Roche, Mannheim, Germany) followed by exposure to a chemiluminescent detection film.

### 2.4. Cell Culture

Human primary chondrocytes were isolated from intact knee cartilage (Outerbridge score 0 or 1) from 6 different donors as described previously [19]. Cells were cultivated in Dulbecco's modified Eagle's medium (DMEM; Sigma-Aldrich, Germany) supplemented with 10% fetal calf serum (FCS), 1% penicillin/streptomycin and 0.1% amphotericin.

### 2.5. Transcriptome Analysis (RNA-Seq)

Prior to RNA-Seq analysis, dose-finding studies were performed, which revealed a concentration-dependent increase in target gene expression (MMP3) from 50 to 250 ng/ml recombinant human PEDF (Biolegend, San Diego, CA) (Suppl. Figure 1 in Supplementary Material available online at https://doi.org/10.1155/2017/7183516). Therefore, we used 250 ng/ml PEDF as the most effective concentration of PEDF for further stimulation experiments.

Articular chondrocytes from 3 different human adult donors were independently stimulated with 250 ng/ml rhPEDF. RNA from stimulated cells and nonstimulated control cells was extracted using the High Pure RNA Isolation Kit (Roche, Mannheim, Germany) according to the manufacturer's protocol. The quality of RNA of PEDF-stimulated cells and control cells was assessed using a 2100 Bioanalyzer system (Agilent Technologies, Santa Clara, CA). Barcoded RNA sequencing libraries were prepared from 100 ng total RNA using TruSeq® Stranded mRNA Sample Preparation Kit, according to the manufacturer's instructions (Illumina, San Diego, CA). To control sources of variation during sample and data processing, a common set of external spike-in RNA controls from Ambion, developed by the external RNA controls consortium (ERCC), was used (Life Technologies, Carlsbad, CA).

Libraries were subjected to single-end sequencing (101 bp) on a HighSeq-2500 platform (Illumina). Quality filtering was performed using cutadapt v. 1.9.1; then reads mapping to rRNAs, tRNAs, snRNAs, and interspersed repeats were filtered out by performing alignment against a manually curated filter reference file using bwa-mem v.0.7.14-r1136 and keeping only unmapped reads. Subsequently, these reads were mapped against the hg19 reference genome using the STAR aligner v. 2.4.0j [20] and a STAR genome directory created by supplying an Ensembl gtf annotation file (genebuild 2013-09) for hg19. Absolute read counts per gene were obtained using Subread's feature Counts program v. 1.4.6-p2 [21] and the Ensembl gtf annotation file.

The following analyses were performed using R version 3.2.1. In particular, differential expression analysis was performed with the DESeq2 package v. 1.8.1 [22]. DESeq2 models count data by means of a negative binomial distribution and employs a Wald test for hypothesis testing. Differences in gene expression were considered statistically significant if their Benjamini-Hochberg adjusted* p* value was less than 0.1. Functional annotation analysis for genes significantly differentially expressed following rhPEDF stimulation and with fold difference in expression between the two sample groups ≥ 2 was performed using Ingenuity Pathway Analysis (IPA, QIAGEN, Redwood City, CA) and Gene Set Enrichment Analysis (GSEA) (Broad Institute; http://www.broadinstitute.org/gsea) [23]. In order to assess robustness of our results, we have reanalyzed our data using edgeR. While DESeq2 identified 1134 differentially expressed genes (adj* p* < 0.1), edgeR identified 1240 differentially expressed genes, 946 of which overlapped between the two analyses. Comparison of the quantile ranks of fold changes revealed a very high correlation between both analytical tools, yielding Spearman's *R*^2^ of 0.94 for all genes and 0.98 for significantly regulated genes (Suppl. Figure 2 a, b).

### 2.6. Quantitative Reverse-Transcription PCR (qRT-PCR)

Quantitative mRNA expression was analyzed in human articular cartilage tissue, periosteal mesenchyme, osteophyte cartilage, and bone. qRT-PCR was also performed in isolated human articular chondrocytes after stimulation with 250 ng/ml rhPEDF. Total RNA was isolated using the High Pure RNA Isolation Kit (Roche Diagnostics, Mannheim, Germany) according to the manufacturer's instructions.

Gene expression levels were quantified by qRT-PCR using the QuantStudio 12K Flex Real-time PCR System and TaqMan RNA-to-Ct 1-Step Kit (Life Technologies, Carlsbad, CA). TaqMan gene expression assays (Applied Biosystems, Foster City, CA) were used for, Serum Amyloid A1 (SAA1) (Hs00761940_s1), interleukin-6 (IL-6) (Hs00174131_m1), matrix metalloproteinase-1 (MMP1) (Hs00233958_m1), matrix metalloproteinase-3 (MMP3) (Hs00233962_m1), matrix metalloproteinase-13 (MMP13) (Hs00233992_m1), vascular endothelial growth factor-A (VEGFA) (forward primer: 5′-GGGCAGAATCATCACGAAGTG-3′; reverse primer: 5′-GGTCTCGATTGGATGGCAGTA-3′; probe 5′-TGAAGTTCATGGATGTCTATCAGCGCAG-3′), sex determining region Y-box 9 (Sox9) (Hs00165814_m1), NKX3-2 (forward primer: 5′-CCAAGAAGGTGGCCGTAAAG-3′; reverse primer: 5′-ACTTCGCCGGGCAGGTAT-3′; probe 5′-TGGTGCGCGACGACCAGAG-3′), aggrecan core protein (ACAN) (Hs00153936_m1), sex determining region Y-box 5 (Sox5) (Hs00374709_m1), chondromodulin-1 (LECT1) (Hs00170877_m1), cartilage oligomeric matrix protein (COMP) (Hs00164359_m1Hs), collagen type II, alpha1 (Col2A1) (Hs00156568_m1), and *β*2-microglobulin (*β*2M) (Hs00984230_m1). The relative quantification of gene expression was performed by the standard curve method. For each sample, the relative amount of the target mRNA was determined and normalized to *β*2M.

### 2.7. Cell Pellet Culture

After passaging, human articular chondrocytes (4 × 10*e*5) from three different donors were cultivated independently at 1% O_2_ in DMEM supplemented with 10 mM *β*-glycerophosphate, 1 nM dexamethasone, and 0.05 mmol/l ascorbate-P-phosphate in monolayer. After 24 h, the cells were stimulated with 250 ng/ml recombinant PEDF. Another 24 h later, the cells were released from monolayer and centrifuged at 2500 rpm for 5 min and left in conical tubes to form pellets. The cells were stimulated again with 250 ng/ml PEDF at day 3 and 7 of pellet culture. The pellets were fixed and stained at day 21 of the pellet culture.

### 2.8. Statistical Analysis

All data are presented as mean ± SD. Statistical analysis was done by GraphPadPrism6 software V 6.04. Quantitative gene expression was analyzed using ANOVA and Student's two-sided* t*-test. All statistical results were considered significant for* p* values < 0.05, unless stated otherwise.

## 3. Results

### 3.1. PEDF Expression in Osteophytes and Different Tissue Types within the Joint

**Figure 1:**
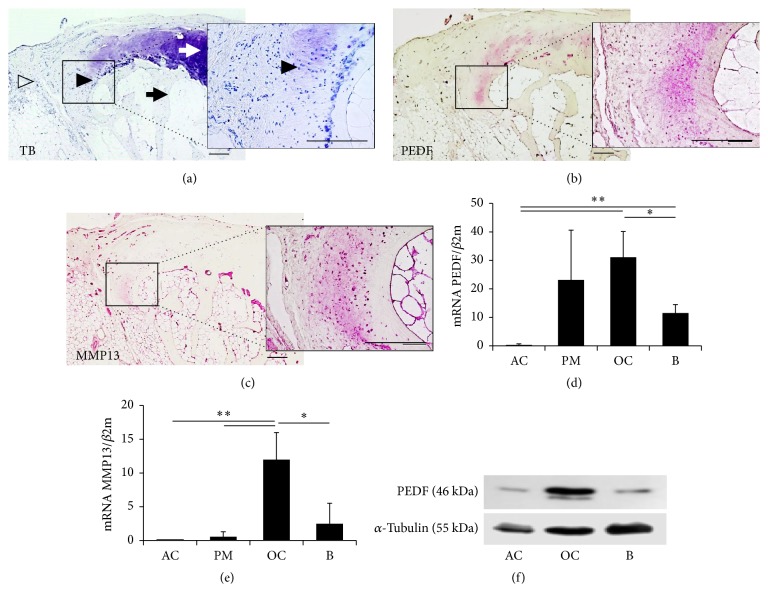
Detection of PEDF mRNA and protein in adult human joint tissues. Toluidine blue staining (a) depicts articular cartilage (white arrow), osteophytic cartilage (black arrowhead), subchondral bone (black arrow), and periarticular mesenchyme (open arrowhead). Inserts show higher magnifications of osteophytic cartilage. PEDF is detectable by immunostaining in osteophytic cartilage and the osteoblastic lining of subchondral bone (b). Correspondingly, MMP13 immunostaining is positive in osteophytic cartilage and subchondral bone (c). Quantitative RT-PCR analysis of PEDF (d) and MMP13 (e) in articular cartilage (AC), periarticular mesenchyme/periosteum (PM), osteophytic cartilage (OC), and subchondral bone (B). Detection of protein levels of PEDF and *α*-tubulin (loading control) by immunoblot analysis (f). Bars = 200 *µ*m. ^*∗*^*p* < 0.05; ^*∗∗*^*p* < 0.01.

The forming osteophyte in osteoarthritic joints represents a model for endochondral ossification within the adult organism. Osteophytes are typically characterized by a superficial fibrocartilaginous layer, followed by a cartilaginous layer and deeper osseous core (Figure 1(a)). Strong immunostaining for PEDF is detectable in the deeper cartilaginous zone of the osteophyte (Figure 1(b)). Periarticular mesenchyme and bone structures show moderate immunoreactivity for PEDF. Articular cartilage is completely negative for PEDF staining (Figure 1(b)). The quantitative gene expression analysis of the corresponding tissues reflects the pattern of immunohistochemistry, with strong PEDF mRNA expression in osteophytic cartilage (OC), bone (B), and periarticular mesenchyme, but virtually no PEDF mRNA expression in healthy articular cartilage (AC) (Figure 1(d)). As a control, the presence and expression of MMP13 were simultaneously analyzed as a reference marker for cartilage tissue undergoing endochondral ossification. The immunohistochemical detection of MMP13 and gene expression analysis revealed strong staining and high mRNA levels within the deeper cartilaginous zones of the osteophyte and moderate expression subchondral bone, but virtually no expression in healthy articular cartilage (Figures 1(c) and 1(e)). Corresponding to the mRNA expression, PEDF protein could hardly be detected by immunoblot in articular cartilage (AC), but strongly in osteophytic cartilage (OC) and moderately in bone tissue (B) (Figure 1(f)).

### 3.2. Detection of PEDF in Fetal Epiphyses and Growth Plate

**Figure 2:**
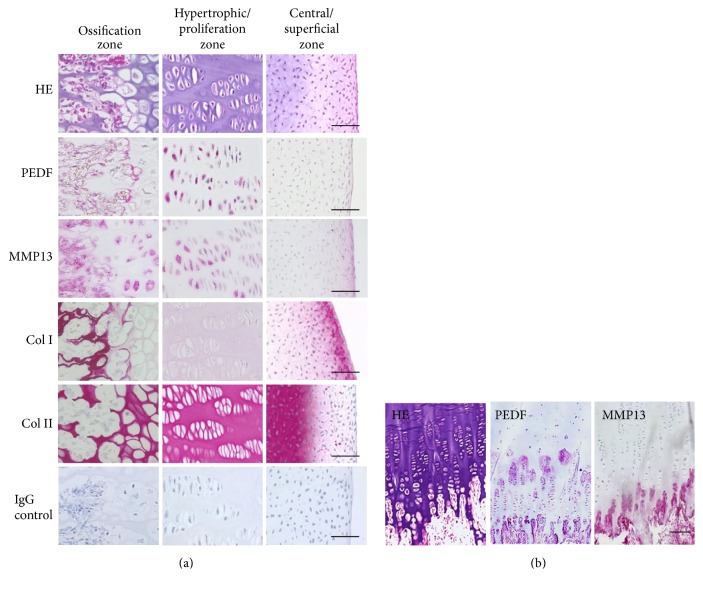
Immunohistochemical analysis of the fetal epiphysis and growth plate. The epiphyses were sectioned into the central and superficial zone, the proliferation and hypertrophic zone, and the ossification zone (a). The typical morphological characteristics of the different zones can be identified by hematoxylin eosin (HE) staining. Immunohistochemical staining for PEDF, MMP13, Col I, and Col II reveals a respective zone-specific pattern. No nonspecific staining was evident in negative controls incubated with isotype normal mouse IgG control. In the postnatal epiphyseal growth plate (b), PEDF is detectable in the proliferation zone, ossification zone, and osteoblastic lining. Correspondingly, MMP13 staining is positive in the hypertrophic zone and osteoblastic lining. Bars = 50 *µ*m.

At the fetal developmental stage, the epiphysis of the femoral condyle is composed of cartilaginous tissue that is characterized by zone-specific cellular phenotypes and still lacks the secondary center of ossification (Figure 2(a)). The superficial zone contains chondrocytes of small diameters at high cellular density. In this zone, PEDF and MMP13 were not detected by immunohistochemistry. The cartilaginous matrix of the superficial zone is typically positive for type II collagen (Col II) and negative for type I collagen (Col I), except for the very superficial cell layer in which this characteristic collagen pattern is inversed. Within the center of the epiphysis, the chondrocytes are surrounded by a type II collagen-positive matrix negative for PEDF and type I collagen immunostaining. MMP13 is not detectable in the epiphyseal center, either. The proliferation zone and ossification zone of the fetal epiphysis resemble the respective zones of the growth plate in later postnatal stages. In the proliferation zone, the chondrocytes are aligned in a columnar pattern and are embedded in matrix positive for type II collagen, PEDF, and MMP13. In the ossification zone, the ossification front and forming bone tissue shows positive immunostaining for type I collagen. PEDF is also strongly detectable in proliferating and hypertrophic chondrocytes as well as in forming bone trabeculae, which corresponds to the immunoreactivity pattern of MMP13.

PEDF and MMP13 can also be detected in the growth plate from a newborn human donor in a zone-dependent manner (Figure 2(b)). While the resting zone was negative for both PEDF and MMP13, moderate staining for PEDF could be detected around the columnar aligned chondrocytes in the proliferation zone. Strong positive staining for both PEDF and MMP13 was found in the lower hypertrophic zone and the osteoblastic lining of forming trabeculae.

### 3.3. Gene Expression Profile and Biological Effects

RNA-Seq data analysis was performed to globally identify potential transcriptional targets of PEDF in primary human chondrocytes. We sequenced the transcriptome of chondrocytes isolated from the knee joints of three individuals that were either stimulated by 250 ng/ml PEDF or left as nonstimulated controls.

**Figure 3:**
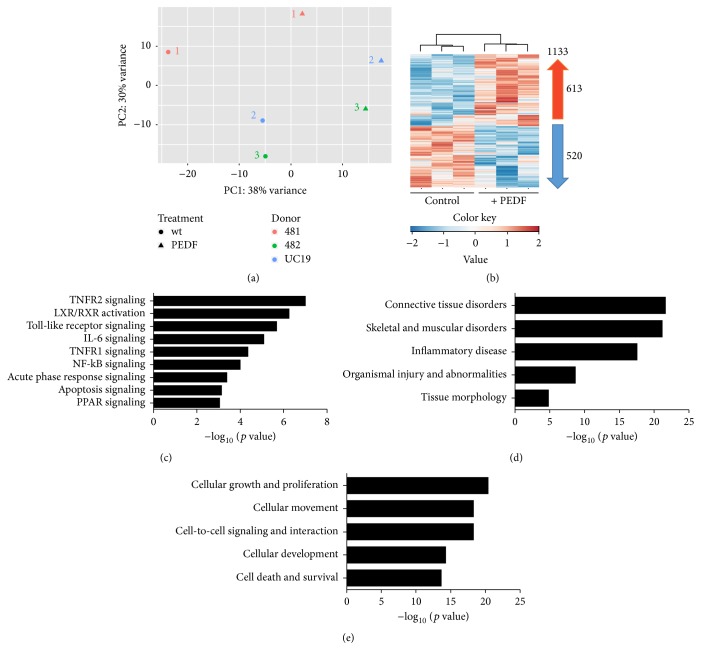
Analysis of PEDF target genes and pathways in chondrocytes using RNA-Seq. Principal component analysis of RNA-Seq expression data demonstrate distinct separation between PEDF-stimulated and nonstimulated chondrocytes (a). Hierarchical clustered heatmap shows differentially expressed genes for each sample. A total of 613 genes (in red) are upregulated and 520 genes (in blue) are downregulated following PEDF treatment (b). IPA-determined enriched signaling pathways (c), disorders (d), and cellular functions (e) associated with PEDF treatment.

The expression patterns of PEDF-stimulated cells and untreated control cells were clearly separated as confirmed by principal component analysis (Figure 3(a)). The hierarchically clustered heatmap depicts a total of 1133 genes that were differentially expressed between chondrocytes stimulated by PEDF and nonstimulated cells using RNA sequencing analysis (adj. *p* < 0.1) (Figure 3(b)). The most differentially expressed genes (>3-fold change) are listed in Table 1. The most significantly upregulated and downregulated genes with roles in the skeletal system are shown in Table 2.

Ingenuity Pathway Analysis (IPA) was applied for the set of genes which were more than 2-fold up- or downregulated by PEDF (adj. *p* < 0.01), in an effort to identify canonical pathways, diseases, and biological functions that are closely related to PEDF stimulus in the context of the skeletal system (Figures 3(c)–3(e)). The top canonical pathways found to be differentially expressed between PEDF-stimulated and nonstimulated control chondrocytes belonged to catabolic, inflammatory, and matrix-degradative pathways (Figure 3(c)). Notably, the most enriched signaling pathways functionally converged to NFkB, as one of the common central elements. The disease and function analysis by IPA demonstrated that the most significantly gene sets influenced by PEDF in chondrocytes were attributable to connective tissue disorders, skeletal and muscular disorders, inflammatory diseases, organismal injury and abnormalities, and tissue morphology (Figure 3(d)). The IPA-analysis for cellular functions enriched by PEDF included cellular growth and development, cellular movement, cell-to-cell signaling, cellular development, and cell death/survival (Figure 3(e)).

**Figure 4:**
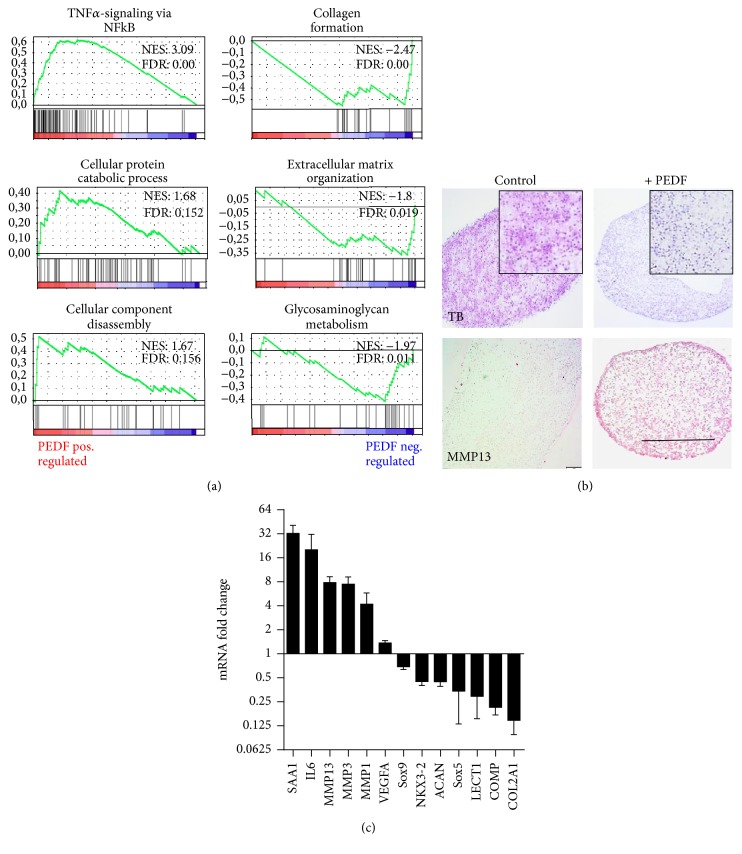
Biological effects exerted by PEDF in chondrocytes. GSEA shows significantly enriched biological processes and signaling pathways based on the gene sets positively and negatively correlated with PEDF treatment (a). PEDF-treated or nontreated three-dimensional cell pellets were analyzed by Toluidine blue (TB) staining and MMP13 immunohistochemistry (b). RNA-Seq data were confirmed by qRT-PCR showing the* n*-fold expression of significantly positively and negatively regulated genes with relevance for the skeletal system (c). Bars show the mean and SD. Bar = 1 mm.

Likewise, GSEA showed significant positive correlation with TNF*α*-signaling via NFkB, cellular protein catabolic processes, and cellular component disassembly, but negative correlation with collagen formation, extracellular matrix organization, and glycosaminoglycan metabolism (Figure 4(a)). These, findings based on transcriptome analysis are in agreement with the biological response of human chondrocytes that were stimulated by PEDF. In three-dimensional pellet cultures, isolated human articular chondrocytes have the capability to redifferentiate and form a proteoglycan-rich matrix, as indicated by a metachromatic Toluidine blue staining and negative staining for MMP13 (Figure 4(b)). In contrast, treatment by PEDF interfered with chondrogenic redifferentiation resulting in a matrix largely lacking proteoglycans. However, the PEDF-treated cell pellets showed a distinct immunoreactivity for MMP13, particularly in the superficial layers (Figure 4(b)).

The data from RNA-Seq were confirmed by qRT-PCR resulting in a 32.5-fold increase of SAA1 mRNA (*p* = 0.003), 7.8-fold increase of MMP13 mRNA (*p* = 0.001), 4.2-fold increase of MMP1 mRNA (*p* = 0.03), 7.5-fold increase of MMP3 mRNA (*p* = 0.003), and 20.2-fold increase of IL6 mRNA (*p* = 0.04) (Table 2; Figure 4(c)). A number of genes involved in skeletal development showed significantly reduced expression as detected by RNA-Seq. These results were confirmed by qRT-PCR, which showed a 7.1-fold decrease of COL2A1 mRNA (*p* = 0.00001), 4.8-fold decrease of COMP mRNA (*p* = 0.00001), and 3.5-fold decrease of LECT1 mRNA (*p* = 0.001) (Table 2; Figure 4(c)). A number of additional cartilage-associated genes were also shown to be significantly downregulated upon PEDF stimulus, including a 2.2-fold decrease of NKX3-2 mRNA (*p* = 0.00004), 2.3-fold decrease of ACAN mRNA (*p* = 0.00006), 2.9-fold decrease of SOX5 mRNA (*p* = 0.01), and a 1.4 decrease in SOX9 mRNA (*p* = 0.001) (Table 2).

## 4. Discussion

This is the first study that comprehensively investigated the role of PEDF on the genome-wide gene expression profile of chondrocytes. We could demonstrate that PEDF significantly upregulates the expression of catabolic and matrix-degrading factors that promote the termination and decay of transient cartilage tissue, while simultaneously, typical cartilage-specific genes are downregulated. SAA1 was the most highly upregulated gene by PEDF. SAA1, an acute-phase protein, was shown to be expressed by chondrocytes and to promote cartilage destruction by stimulating the expression of matrix metalloproteinases [24]. SAA1 is also expressed by MSCs and osteoblasts, in which it may support the proinflammatory phase of osteogenic differentiation and enhanced mineralization [25, 26].

Interestingly, MMP13, the reference marker for terminal chondrocyte differentiation and endochondral ossification, was among the 5 most significantly upregulated genes following PEDF stimulus. Immunohistochemical analysis demonstrated a corresponding staining pattern of MMP13 to that of PEDF, which was observed in the zone of endochondral ossification in human epiphyseal cartilage, growth plate cartilage, and osteophytic tissue. MMP13 is the major collagenase expressed in the primary and secondary ossification centers and drives endochondral ossification by degrading major cartilage components, such as type II collagen and aggrecan [27]. MMP13 is known as one crucial mediator to promote the endochondral ossification process and the terminal differentiation of chondrocytes [27, 28]. MMP13 is also required for adequate bone development, in particular bone remodeling [29]. Thus, MMP13 may be one of the most relevant target genes induced by PEDF for the skeletal system, in particular for cartilage tissue that undergoes endochondral ossification.

PEDF is a pluripotency protein that is expressed in multiple tissues. Several signaling pathways have been described for PEDF, including the IP3-AKT, MEK-ERK, or PLA_2_-PPAR*γ* pathway, which all converge in the activation of the NF*κ*B complex [30–34]. Indeed, the Ingenuity Pathway Analysis IPA of the present study revealed NF*κ*B as one common central target upon PEDF stimulus. In fact, NF*κ*B- and ERK-pathways are known to be the main mechanisms that induce MMP13 and MMP1 expression in chondrocytes [35–37].

The activation of the NF*κ*B and PPAR*γ* pathways may also explain the highly significant downregulation of many genes encoding for cartilage-specific matrix proteins. Most of these genes are under the transcriptional control of the transcriptional complex of Sox5, Sox6, and Sox9 [38]. Sox9 is a central transcription factor for a large number of cartilage-specific genes and represents stabilizing factor of the chondrocyte phenotype [38]. The only moderate downregulation of Sox5 and Sox9 gene expression by PEDF may not solely explain the highly significant downregulation of many cartilage genes. It is likely that posttranscriptional mechanisms contribute to the antichondrogenic effects of PEDF. In this context, the activated NF*κ*B complex and PPAR*γ* were shown to reduce the transcriptional activity of Sox9 by interfering with nuclear translocation, destabilization of Sox9 mRNA, and limiting the availability of cofactors such as p300 [39–41]. Furthermore, MMP13 itself may affect the activity of Sox9, since knock-down of MMP13 resulted in increased nuclear translocation of Sox9, [42, 43].

Recently, the loss of PEDF function was identified to be associated with osteogenesis imperfecta type VI that is characterized by a severe skeletal phenotype. In human mesenchymal stem cells, PEDF was able to induce osteoblastic related genes such as ALP, Runx2, OCN, and BSP and increase ALP activity and support mineral deposition, albeit, this effect was much lower than the effect achieved by osteogenic medium [16]. Nevertheless, an in vivo model demonstrated that PEDF is able to mediate ectopic bone formation in muscle pockets [44]. In the present study focusing on the gene expression of chondrocytes, we could not detect significant effects of PEDF on the expression of osteogenic genes. Thus, the impact of PEDF on endochondral ossification may rather be based on degradation and remodeling processes than on induction of mineral deposition. However, further work is needed to explore the impact of PEDF on mineral deposition in cells other than mesenchymal stem cells, such as matrix-embedded chondrocytes. Indeed, high-resolution backscattered electron imaging and synchrotron X-ray scattering of human bone biopsies of osteogenesis imperfecta type VI patients revealed even an increased calcium content of the bone matrix, as well as coexistence of highly mineralized bone matrix with seams of abnormally low mineral content atypical collagen fibril organization around osteocytes, which supports the concept of a disturbed matrix remodeling during early steps of mineralization [15]. In fact, a similar phenotype could be observed in MMP13-deficient mice which are characterized by an impaired remodeling of the perilacunar matrix, disruption of the canalicular network, abnormal collagen, and mineral organization [29]. These observations underline the assumption that PEDF exerts its functional role in endochondral ossification via MMP13.

PEDF could also be detected within cartilage repair tissue induced by bone marrow-stimulating techniques (data not shown). The repair tissue typically suffers from an instable transient chondrocyte phenotype and excessive subchondral bone formation within the defects [45–47]. This supports the concept that PEDF serves as a marker for a transient chondrogenic differentiation status and the corresponding staining pattern of MMP13 (data not shown) may serve as a trigger for inadvertent endochondral ossification within cartilage repair tissue.

In conclusion, PEDF plays an important role in the endochondral ossification process during all developmental stages. PEDF mediated the decay of transient cartilage tissue by promoting matrix degradation and suppression of cartilage-specific gene expression. PEDF represents a marker for the transient status of the chondrocyte phenotype that is subjected to terminal differentiation and endochondral ossification. The inhibition of PEDF action may be the basis for future therapeutic strategies in order to stabilize the chondrocyte phenotype of articular cartilage and to prevent its degradation.

## Supplementary Material

Suppl. Figure 1: Prior to RNA-Seq analysis, dose finding studies were performed. Articular chondrocytes, cultured in monolayer, were stimulated with 50, 100, and 250 ng/ml recombinant human PEDF. Target gene expression (MMP3) was detected by quantitative RT-PCR analysis. Stimulation by 250 ng/ml PEDF showed the most striking increase in target gene expression (7.5-fold; P = 0.003). This concentration was used for further stimulation experiments. Suppl. Figure 2 a,b: In order to assess robustness of our results, we have reanalyzed our data using edgeR. While DESeq2 identified 1134 differentially expressed genes (adj p < 0.1), edgeR identified 1240 differentially expressed genes, 946 of which overlapped between the two analyses. Comparison of the quantile ranks of fold changes revealed a very high correlation between both analytical tools, yielding a Spearman's R^2^ of 0.94 for all genes and 0.98 for significantly regulated genes

## Figures and Tables

**Table 1:** Most differentially expressed genes in PEDF-stimulated cells versus nonstimulated control cells (change > 3-fold).

Gene symbol	Description	Fold change	Adj *p*
SAA1	Serum amyloid A1	34.58	<1.0*E* − 120
NOS2	Nitric oxide synthase 2, inducible	20.13	4.89*E* − 118
LCN2	Lipocalin 2	15.97	<1.0*E* − 120
C3	Complement component 3	12.00	3.22*E* − 171
MMP13	Matrix metallopeptidase 13 (collagenase 3)	10.46	5.59*E* − 93
MT1H	Metallothionein 1H	10.39	1.63*E* − 56
HSD11B1	Hydroxysteroid (11-beta) dehydrogenase 1	10.02	2.28*E* − 90
LTF	Lactotransferrin	8.94	1.62*E* − 53
PTX3	Pentraxin 3, long	8.92	4.89*E* − 118
CXCL5	Chemokine (C-X-C motif) ligand 5	8.86	7.08*E* − 29
VNN2	Vanin 2	8.33	1.31*E* − 32
BIRC3	Baculoviral IAP repeat containing 3	7.73	3.05*E* − 49
CXCL2	Chemokine (C-X-C motif) ligand 2	7.33	6.25*E* − 24
MT1F	Metallothionein 1F	7.25	2.15*E* − 48
SOD2	Superoxide dismutase 2, mitochondrial	7.06	5.50*E* − 164
PI3	Peptidase inhibitor 3, skin-derived	6.82	3.28*E* − 22
MT1G	Metallothionein 1G	6.75	3.79*E* − 23
CCL2	Chemokine (C-C motif) ligand 2	6.50	2.98*E* − 107
IL32	Interleukin 32	5.85	8.08*E* − 19
VCAM1	Vascular cell adhesion molecule 1	5.61	1.31*E* − 66
IL4I1	Interleukin 4 induced 1	5.49	4.38*E* − 18
LAMB3	Laminin, beta 3	5.46	7.05*E* − 99
CX3CL1	Chemokine (C-X3-C motif) ligand 1	5.43	7.54*E* − 28
TNFAIP2	Tumor necrosis factor, alpha-induced protein 2	5.31	5.42*E* − 51
TLR2	Toll-like receptor 2	4.99	8.76*E* − 58
CST2	Cystatin SA	4.62	2.95*E* − 14
CHI3L2	Chitinase 3-like 2	4.44	2.82*E* − 68
SLC7A2	Solute carrier family 7 (cationic amino acid transporter), member 2	4.37	2.65*E* − 72
RELB	V-rel avian reticuloendotheliosis viral oncogene homolog B	4.05	2.58*E* − 24
IL34	Interleukin 34	3.86	3.32*E* − 18
CSF1	Colony stimulating factor 1 (macrophage)	3.82	1.62*E* − 53
TNFAIP8	Tumor necrosis factor, alpha-induced protein 8	3.77	1.17*E* − 17
WTAP	Wilms tumor 1 associated protein	3.71	5.00*E* − 47
VNN3	Vanin 3	3.66	3.56*E* − 10
ELF3	E74-like factor 3 (ETS domain transcription factor, epithelial-specific)	3.60	1.61*E* − 11
RARRES1	Retinoic acid receptor responder (tazarotene induced) 1	3.56	1.26*E* − 10
NFKBIA	Nuclear factor of kappa light polypeptide gene enhancer inhibitor, alpha	3.53	2.20*E* − 41
MMP1	Matrix metallopeptidase 1 (interstitial collagenase)	3.50	1.32*E* − 10
NOD2	Nucleotide-binding oligomerization domain containing 2	3.45	1.37*E* − 09
LBP	Lipopolysaccharide binding protein	3.29	2.40*E* − 20
LIF	Leukemia inhibitory factor	3.25	1.40*E* − 15
CD74	CD74 molecule, major histocompatibility complex, class II invariant chain	3.13	1.41*E* − 21
TNFAIP3	Tumor necrosis factor, alpha-induced protein 3	3.13	1.63*E* − 18
MT1E	Metallothionein 1E	3.09	7.77*E* − 24
IRAK2	Interleukin-1 receptor-associated kinase 2	3.07	4.03*E* − 24
MSC	Masculine	3.06	7.48*E* − 08
PLK2	Polo-like kinase 2	3.05	3.82*E* − 17
SLC30A2	Solute carrier family 30 (zinc transporter), member 2	3.04	2.15*E* − 07
G0S2	G0/G1switch 2	3.00	3.86*E* − 18
OGN	Osteoglycin	−3.03	3.96*E* − 34
COMP	Cartilage oligomeric matrix protein	−3.05	9.37*E* − 48
ACTC1	Actin, alpha, cardiac muscle 1	−3.07	2.01*E* − 10
CFH	Complement factor H	−3.07	1.65*E* − 57
COL9A3	Collagen, type IX, alpha 3	−3.14	8.30*E* − 65
COL9A2	Collagen, type IX, alpha 2	−3.18	1.32*E* − 46
SLC14A1	Solute carrier family 14 (urea transporter)	−3.28	7.13*E* − 14
ADAMTSL2	ADAMTS-like 2	−3.34	9.68*E* − 17
CHAD	Chondroadherin	−3.92	3.85*E* − 30
COL2A1	Collagen, type II, alpha 1	−4.33	3.10*E* − 49
CYTL1	Cytokine-like 1	−4.45	2.11*E* − 35
GDF10	Growth differentiation factor 10	−4.57	1.43*E* − 46

**Table 2:** Selected genes with roles in the skeletal system significantly up- or downregulated by rhPEDF.

gene symbol	Description	Fold change	Adj *p*	Ranking among the upregulated genes (*n* = 613)	qRT-PCR	
					Fold change mean (95% CI)	*P* value
SAA1	Serum amyloid A1	34.53	<1.0*E* − 120	1	32.5 (11.5–53.5)	0.003
NOS2	Nitric oxide synthase 2, inducible	20.11	4.89*E* − 118	2		
MMP13	Matrix metallopeptidase 13	10.41	5.59*E* − 93	5	7.8 (4.3–11.3)	0.001
SOD2	Superoxide dismutase 2, mitochondrial	7.06	5.50*E* − 164	15		
TLR2	Toll-like receptor 2	4.99	8.76*E* − 58	25		
MMP1	Matrix metallopeptidase 1	3.50	1.32*E* − 10	38	4.2 (0.2–8.2)	0.03
MMP3	Matrix metallopeptidase 3	2.28	2.20*E* − 4	87	7.5 (3.2–11.7)	0.003
PPARG	Peroxisome proliferator-activated receptor gamma	1.65	0.031	275		
ADAMTS1	ADAM metallopeptidase with thrombospondin type 1 motif, 1	1.55	7.48*E* − 07	338		
NFKB1	Nuclear factor of kappa light polypeptide gene enhancer in B-cells 1	1.53	1.70*E* − 4	345		
IL6	Interleukin 6 (interferon, beta 2)	1.47	0.076	386	20.2 (−7.7–48.2)	0.04

SOX9	SRY (sex determining region Y)-box 9	−1.31	6.30*E* − 04	370	−1.4 −(1.2–1.8)	0.001
SOX5	SRY (sex determining region Y)-box 5	−1.61	1.02*E* − 05	143	−2.9 −(1.2–5.5)	0.01
ACAN	Aggrecan	−1,75	4.49*E* − 08	104	−2.3 −(1.7–3.2)	0.00006
COL9A1	Collagen, type IX, alpha 1	−1.82	7.93*E* − 08	84		
NKX3-2	NK3 homeobox 2	−1.92	9.43*E* − 05	66	−2.2 −(1.7–3.0)	0.00004
FGFR3	Fibroblast growth factor receptor 3	−1.92	2.51*E* − 10	53		
S100A1	S100 calcium binding protein A1	−2.17	1.43*E* − 22	41		
PRG4	Proteoglycan 4	−2.27	2.01*E* − 26	29		
LECT1	Leukocyte cell derived chemotaxin 1	−2.33	5.31*E* − 07	26	−3.5 −(1.6–18.5)	0.001
COL11A1	Collagen, type XI, alpha 1	−2.38	1.68*E* − 24	25		
COL11A2	Collagen, type XI, alpha 2	−2.50	1.71*E* − 24	20		
FRZB	Frizzled-related protein	−2.87	6.31*E* − 27	14		
COMP	Cartilage oligomeric matrix protein	−3.03	9.37*E* − 48	11	−4.8 −(3.2–9.2)	0.00001
COL9A2	Collagen, type IX, alpha 2	−3.12	1.32*E* − 46	7		
COL9A3	Collagen, type IX, alpha 3	−3.13	8.30*E* − 65	8		
CHAD	Chondroadherin	−4.00	3.85*E* − 30	4		
COL2A1	Collagen, type II, alpha 1	−4.32	3.10*E* − 49	3	−7.1 −(3.8–41.4)	0.00001
