# Supplementary material for: PEDF Is Associated with the Termination of Chondrocyte Phenotype and Catabolism of Cartilage Tissue
Source: Biomed Res Int. 2017 Jan 16;2017:7183516. doi: 10.1155/2017/7183516 (PMC5278211; doi:10.1155/2017/7183516)
Supplement: Supplementary file 1 — Suppl. Figure 1: Prior to RNA-Seq analysis, dose finding studies were performed. Articular chondrocytes, cultured in monolayer, were stimulated with 50, 100, and 250 ng/ml recombinant human PEDF. Target gene expression (MMP3) was detected by quantitative RT-PCR analysis. Stimulation by 250 ng/ml PEDF showed the most striking increase in target gene expression (7.5-fold; P = 0.003). This concentration was used for further stimulation experiments. Suppl. Figure 2 a,b: In order to assess robustness of our results, we have reanalyzed our data using edgeR. While DESeq2 identified 1134 differentially expressed genes (adj p < 0.1), edgeR identified 1240 differentially expressed genes, 946 of which overlapped between the two analyses. Comparison of the quantile ranks of fold changes revealed a very high correlation between both analytical tools, yielding a Spearman's R2 of 0.94 for all genes and 0.98 for significantly regulated genes [file 7183516.f1.pdf]

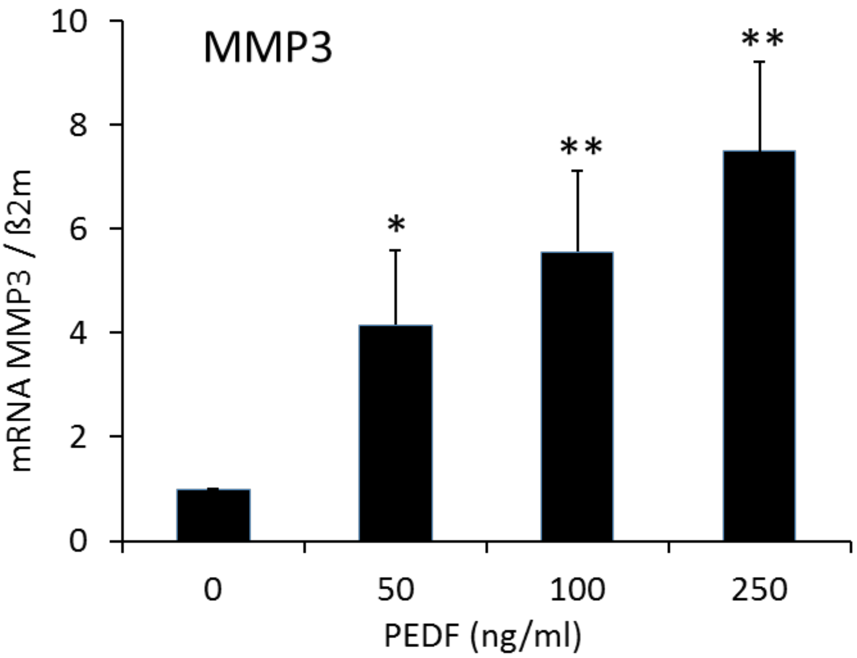

Supplementary Figure 1:

Quantitative RT-PCR analysis of MMP3 mRNA expression following treatment by different concentrations of PEDF. *P < 0.05; **P < 0.01

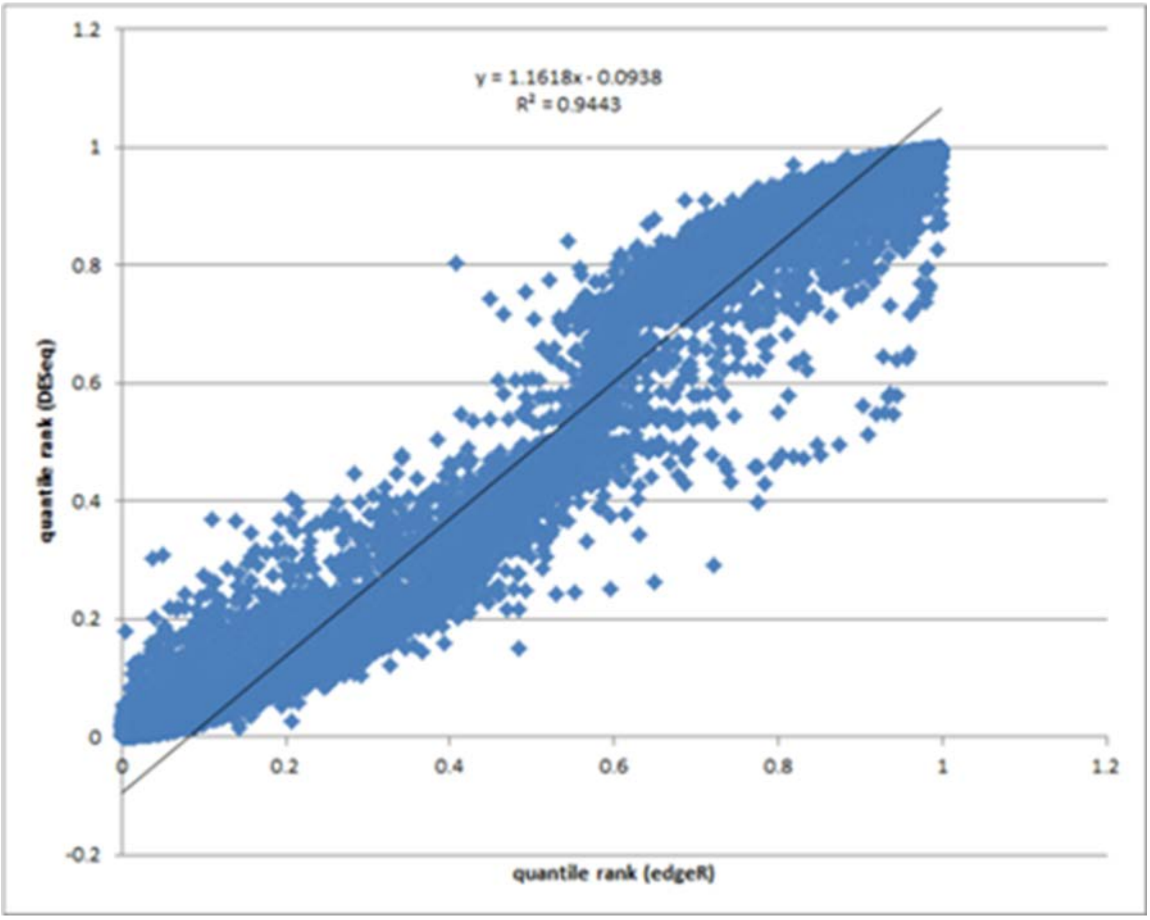

Supplementary Figure 2 a:

Correlation between edgeR and DESeq2 for all genes

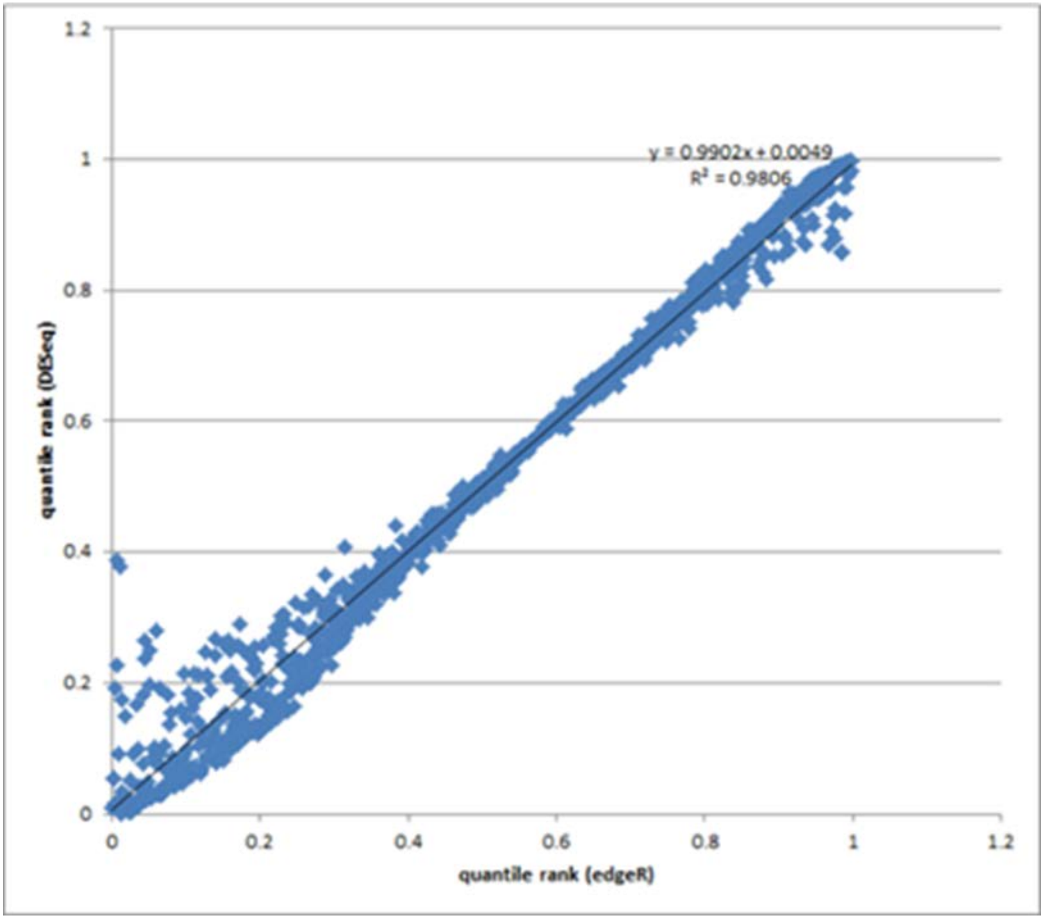

Supplementary Figure 2 b:

Correlation between edgeR and DESeq2 for significantly regulated genes (adjp<0.1, n=946)
